# Prof. M. M. Mukherjee

**DOI:** 10.4103/0970-0358.63939

**Published:** 2010

**Authors:** Sasanka Sekhar Chatterjee

**Affiliations:** Department of Plastic Surgery, Institute of Post Graduate Medical Education and Research, Kolkata, India E-mail: ssc_dr2004@yahoo.co.uk

**Figure:**
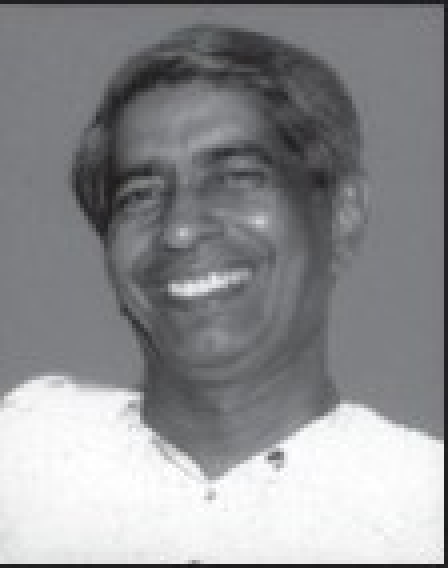
Prof. M. M. Mukherjee

Prof. Murari Mohan Mukherjee was a legend of a Plastic Surgeon for more reasons than one. Whether it was his understanding of tissue behavior and gentle handling or his ability to predict the final outcome of a procedure and communicate the same to his patients and students alike or his up-to-date knowledge and the desire to continuously improve his performance as a surgeon and a teacher, Bengal and India will never have his match.

Murari Mohan Mukherjee was born on December 30, 1914, in Bhagalpur, Bihar, in his maternal uncle's house. He was related to the great litterateur Rishi Bankim Chandra Chattopadhyay through his maternal lineage. His native place was, however, in Chuchura in Hooghly district of West Bengal. After his matriculation with district scholarship, in 1931, he obtained 'Duff' scholarship during his intermediate course in Presidency College, Calcutta, which was undoubtedly the best institution in India at that time. He completed MBBS from Medical College, Calcutta, in 1939, having won 11 gold and eight silver medals in his career. In the next 13 years he served in the Government health service and did his MS in General Surgery from Calcutta in 1949. The next two years were spent in England in association with Sir Harold Gillies, A.B Wells and Kilner. Having completed FRCS from England and Edinburgh, he returned to Calcutta in November, 1951.

From 1952 onwards, he began performing Plastic surgical operations as a resident medical officer in General Surgery at the Presidency General hospital, Calcutta, now known as IPGME&R. A separate plastic surgical unit was created in 1954 in PG hospital and his fame had reached far and wide to draw the attention of the then CM late Bidhan Chandra Roy. In 1956, a department was opened in the same hospital. It was inaugurated by no less a person than Pandit Nehru and Professor Benjamin Rank of Australia graced the occasion with Dr. B. C. Roy presiding over it.

He was very fond of mathematics and liked to solve puzzles and problems This very nature of his drove him to plastic surgery, which is now called problem solving surgery. He was excited by patients presenting with various problems which could ignite his imaginative capabilities with intellectual satisfaction. Added to that was his empathy for patients; he always believed that something can be done. He was an embodiment of discipline, diligence and patience. However tight his schedule would be, he would always worship God before leaving his house in the morning. In his own words "I always pray to god before the start of the day that no harm should occur to the patient due to my lapses of concentration or negligence". Despite performing 8 to 10 operations a day, he was always tireless and had an infectious smile. He had a very busy practice yet he always entered the hospital in time, made it a point to see all his hospital patients daily and always visited the operated patients of the Government hospital in the evening after his private practice of the day was over. All patients were treated equally, be it in Private, Government or Trust hospitals. There was not a single contemporary subject in plastic surgery he was not adept in and always performed even the newest known operations with finesse and élan. His presentations in World Congresses, references in Cleft Craft and invited articles in 'Clinics in Plastic Surgery' speak volumes of his academic contribution.

He never considered any operation in plastic surgery difficult and would spend time planning an operation till the end with great attention to details so that he completed the operation mentally before its start. However, despite a plan, quick decisions to switch over came naturally to him. It was in one such situation when a quick closure was necessary for a cheek defect during an oncologic resection for carcinoma cheek that he had planned a tongue flap for the inner lining – an instant decision but how expedient!! Similarly the birth of buccal mucosal flap for cleft palatoplasty, which used to be called the cheek flap then. After so many years, we have realized that it has been used much less often than it deserves.

He was the Head of the Unit/Department from 1955 to 1972 and an emeritus professor. Between 1965 and 1968; he was additionally the Surgeon Superintendent and Director of IPGME&R. Despite this great responsibility there was no let up in his surgical work. As a surgeon superintendent he brought about lot of changes with regard to patient care, improvement of working conditions of employees including formation of their club, public relations system and established a vehicular covered transport for the corpses instead of on open trolleys. There was also a place for prayer for relatives of the deceased as per their religion. During this time, he established a separate burn unit. His philanthropic nature and attachment to the roots was exhibited much before he became an established plastic surgeon, when in a plot of land in front of his native house, he had established a library of text books 'Kishore Pragati Sangha'and a health center for the economically ward people of the region. This same nature lured him to establish a center for the physically challenged in hearing and speech in Hooghly, a place about 70 km from Kolkata and 'Rehabilitation India' for the physically challenged of all types in Kolkata itself. He also donated many instruments and created an operation theatre exclusively for plastic surgery named Shyampukur Pallimangal Samiti in North Kolkata where he had lived for some time. This was all for the poor and needy. This great man breathed his last on July 26, 1988.

